# Follow-up after gestational diabetes: a qualitative study of perspectives from general practices

**DOI:** 10.3399/BJGPO.2021.0241

**Published:** 2022-07-27

**Authors:** Jane Hyldgaard Nielsen, Kirsten Fonager, Jette Kolding Kristensen, Charlotte Overgaard

**Affiliations:** 1 Public Health and Epidemiology Group, Department of Health Science and Technology, Aalborg University, Aalborg, Denmark; 2 DECIPHer, Cardiff School of Social Sciences, Cardiff University, Cardiff, UK; 3 Clinical Nursing Research Unit, Aalborg University Hospital, Aalborg, Denmark; 4 Midwifery, University College of Northen Denmark, Aalborg, Denmark; 5 Department of Social Medicine, Aalborg University Hospital, Aalborg, Denmark; 6 Department of Clinical Medicine, Aalborg University, Aalborg, Denmark; 7 Center for General practice, Aalborg University, Aalborg, Denmark

**Keywords:** diabetes, gestational, diabetes mellitus, type 2, follow-up screening, general practice, disease prevention, qualitative health research, primary healthcare

## Abstract

**Background:**

Women whose pregnancies are complicated by gestational diabetes mellitus (GDM) are approximately eight times more likely to develop type 2 diabetes mellitus (T2DM). Although regular participation in follow-up screening increases the chance of early detection of diabetes, participation rates are often suboptimal. A better understanding of general practice as a key contextual setting for screening could help inform the development and adoption of, for example, electronic reminder interventions to support women’s participation.

**Aim:**

To explore the perspectives of GPs and relevant staff members engaged in early detection of diabetes after gestational diabetes in order to identify barriers to and facilitators of follow-up screening.

**Design & setting:**

A qualitative interview study undertaken in general practices in the North Denmark Region.

**Method:**

Based on a purposive sample strategy, 18 semi-structured interviews of 12 GPs and six staff members, who were either nurses or midwives, were analysed using a reflexive thematic analytical approach.

**Results:**

The following three main themes were formulated: (1) challenges of addressing women’s risk; (2) prioritisation of early detection of diabetes; and (3) system influence on clinical procedures.

**Conclusion:**

Follow-up screening was facilitated by knowledge of guidelines. Professional collaboration and adaptation support were found to lead to successful implementation of guidelines in general practice. Supporting GPs and their staff’s reflection and discussion of ways to engage in communication and decisionmaking processes with women is, however, important; it was found to create an important barrier to follow-up screening. Overall, the findings can help inform the future development of interventions to increase uptake.

## How this fits in

Participation rates in the recommended follow-up screening for T2DM after birth are often suboptimal. The effect of electronic reminder interventions to support uptake varies with contextual factors. Exploring the experiences of individuals working in general practice may contribute to the development and adoption of future interventions such as reminders. By including the perspectives of both GPs and staff responsible for follow-up screening, this study provides new insights into both facilitators of and barriers to screening.

## Introduction

In Denmark, approximately 3%–4% of pregnancies are affected by GDM.^1^ Close to one-third of women diagnosed with GDM develop T2DM within 15 years.^2^ This group of generally otherwise healthy women are thus at high risk for T2DM at an early age, although hyperglycaemia usually passes soon after birth.^3,4^

Regular and recurrent participation in follow-up screening after GDM provides an important opportunity for lifestyle interventions and early detection of T2DM.^5,6^ However, suboptimal follow-up screening presents a challenge across healthcare systems.^7–9^ A previous study has found participation rates of approximately 17% at 4–6 years after birth.^7^

The responsibility for follow-up care for women after GDM is often unclear to health professionals, although it is generally placed with general practices.^10^ Practical family related issues affect women’s motivation and ability to attend, but factors related to the specific healthcare system, such as clinicians’ attitudes, poor continuity of care, and test-booking procedures have also been shown to exert an important influence.^11–13^ Furthermore, studies of clinicians involved in follow-up screening have identified barriers concerning the communication of diagnoses, transition of care, and competing clinical priorities.^14–17^ Clinicians’ knowledge, attitudes, and beliefs concerning follow-up screening may likewise be of significance.^18^

International studies indicate that screening support systems, such as electronic reminders to either women with prior GDM or their GPs, can help bridge cross-sectoral transitions and increase participation.^19–22^ Although they may seem a straightforward measure, few interventions, such as electronic reminders, are in fact truly simple.^23^ As shown by a systematic review of variations in their effect,^19^ reminders should be carefully adjusted to the local context. Each context has its unique characteristics and circumstances that interact, modify, facilitate, or constrain the delivery and the effect of interventions, including differences in starting points, conflicting norms and/or insufficient resources.^24^ A better understanding of general practice as a key contextual setting for screening could help elucidate the potential of reminder-based interventions. It could furthermore help generate new knowledge on how reminders create change and thereby inform the development and adoption of optimal reminders. This could improve the organisation of care for women with previous GDM.

This study aimed to explore the perspectives of GPs and relevant staff members (that is, registered nurses and midwives) on follow-up screening for T2DM after GDM, and to identify barriers to and facilitators of follow-up screening.

## Method

### Design

A qualitative study based on in-depth, semi-structured interviews. The reporting was guided by the recommendations of the Standards for Reporting Qualitative Research (SRQR) checklist.^25^

### Setting

The study was conducted during the development phase of an intervention study to support follow-up screening of women with previous GDM. Data were collected between March and December 2019 in the North Denmark Region, one of the five Danish regional authorities governing both primary and secondary healthcare services.^26^ The Danish welfare model offers universal health care to promote society-wide health and social equity,^26^ an endeavour in which general practices have a key position.^26^ The population of the region was approximately 0.6 million people.^26^ Each of the region’s 285 GPs served on average 1779 patients.^27^ Most general practices were private solo or group practices, while a few were managed by the region administration.^26^ Danish guidelines urge women with prior GDM to undergo an oral glucose tolerance test (OGTT) at 12–16 weeks postpartum and an HbA1c test every 1–3 years thereafter.^28^ To support adherence, some women may receive a prescheduled appointment to the first screening months after birth but not for the subsequent screening test; all offered free of change by their GP.^7^ Women may also be offered free support for lifestyle change by their GP or delegated staff or participation in interventions organised by the municipality.

### Participant selection

A purposeful sampling strategy was adopted to allow for a detailed exploration and understanding of the topic.^29^ All general practices in the region were eligible for inclusion and received an open invitation to participation in a newsletter from the regional general practice research unit. It was aimed to include a mixed sample representing solo and group practices in both urban and rural areas, with participants of different ages, sex, and experience.^29^ As the open invitation elicited no response, general practices were selected by direct phone or personal contact. Some GPs choose to be represented by a staff member with delegated responsibility for pregnant women and/or follow-up care for patients with diabetes or other chronic diseases.

### Data collection

The first author conducted the semi-structured interviews, which all were audio-recorded and finally transcribed verbatim by an assistant. Three of the included participants preferred telephone interviewing, while the study opted for in-person interviews, as they enable direct observation of emotional and visual cues of importance to the dynamics, interpretations, and depth of the interview.^30^ A recent study has found in-person interviews to be marginally superior, although the difference was neglegible.^31^ This also applied for the present study. All interviews were undertaken in general practice during the clinics’ working hours and lasted about 45–70 minutes. As a pilot test prompted only very minor changes and refinements to the interview guide, the original interviews were accepted as study material.^30^

### Data analysis

To identify general patterns in the data, themes were conceptualised as analytic output according to reflexive thematic analysis inspired by Braun and Clarke.^32^ The reflexive notes taken during the first reading of the transcribed interviews were discussed among the authors. Using NVivo qualitative data analysis software (version 12), the entire dataset was subsequently inductively coded, for both semantic and latent meanings.^32^ To stimulate reflexivity and coding rigour, the transcripts, codes, and emerging themes were compared and discussed by two of the researchers. The entire group of researchers were involved in discussion of the definition and revision of themes.

### Ethical considerations

As Danish legislation holds qualitative studies to be based solely on the participants’ informed written consent, no ethical approval was required.^33^ However, the study followed the research ethics recommendations of the Danish Ministry of Higher Education and Science^34^ and the Declaration of Helsinki.^35^ Before data were collected, the participants were informed about the study’s purpose and its policies of data protection and storage, privacy and confidentiality. It was ensured that the participants understood that they could withdraw consent at any time.

## Results

Eighteen participants, comprising 12 GPs and six staff members, accepted the invitation to participate. Their data are shown in Table 1.

**Table 1.** Participants’ key characteristics

Participant	Area	Sex	Age range, years	Profession	Range of years in general practice
A	Urban	Female	40–45	GP	5–10
B	Urban	Female	40–45	GP	<5
C	Rural	Female	45–50	GP	15–20
D	Rural	Female	50–55	GP	15–20
E	Rural	Male	60–65	GP	25–30
F	Urban	Female	40–45	GP	<5
G	Urban	Female	40–45	GP	5–10
H	Urban	Male	65–70	GP	25–30
I	Rural	Female	45–50	GP	5–10
J	Rural	Female	40–45	GP	<5
K	Urban	Female	60–65	GP	25–30
L	Rural	Female	40–45	GP	5–10
M	Rural	Female	30–35	Nurse or midwife	<5
N	Rural	Female	40–45	Nurse or midwife	<5
O	Rural	Female	30–35	Nurse or midwife	<5
P	Rural	Female	55–60	Nurse or midwife	15–20
Q	Rural	Female	40–45	Nurse or midwife	<5
R	Rural	Female	35–40	Nurse or midwife	5–10

The key meanings expressed during the interviews were analysed and condensed into three main themes and five sub-themes, which are shown in Table 2.

**Table 2.** Themes and sub-themes

Themes	Sub-themes
Challenges of addressing women’s risk	*Insufficient knowledge* *Balancing contradicting risk perceptions*
Prioritisation of early detection of diabetes	
System influence on clinical procedures	*Systematising clinical procedures to improve quality of care* *Trusting own clinical skills without system interference* *Influence of recommended test*

### Theme 1: Challenges of addressing women’s risk

#### Insufficient knowledge

Overall, many participants had sparse knowledge of the increased risk of T2DM after birth, which led to a rather weak focus. Although this was most noticeable among the GPs, it was true also for other staff members:

*'I have to say, I actually feel lucky that you called …* [Otherwise,] *our patients would have fallen between the cracks for a long time. Because we had no idea.'* (Q)

The staff participants linked the poor focus to a lack of general knowledge in their practice concerning women with previous GDM. In those general practices where staff were aware of women’s much higher risk, they followed clear instructions given by the GP or relied on interdisciplinary discussion and knowledge sharing in their clinic.

While some GPs were aware of women’s increased risk, they had no clear conception of recommendations concerning times for screening, testing, or the benefits of screening in general. In those cases, the insufficient knowledge was often associated with a hesitant or unconcerned approach to screening. The GPs tended to underestimate women’s risk and expressed their surprise at the seriousness of this during interviews. Overall, the participants often linked their underestimation of risk for T2DM to the women’s youth:

*'I’m wondering … they’re young women and they don’t fit into our understanding of being at risk. I mean … I know it’s because they had GDM, but I think we could cut them some slack? … Their risk can’t possibly be that high?'* (L)

This was the dominant view, which seemed to strengthen or rationalise the reluctant approach to the random screening, which was frequently offered only on the women’s insistence.

The participants also emphasised that screening measures were compromised by the maternity ward’s discharge summaries:

*'It’s really, really important that, instead of such a ridiculous discharge summary from the maternity ward stating all those silly diagnostic codes … It should be simplified so that it says, “You need to be followed-up by your own GP”* [stating clearly that the GP] *must follow-up on this, that or the other …'* (F)

The inadequate transfer of information and compromised communication lines between healthcare sectors seemed to create significant barriers, and the general practices found it challenging to stay updated.

#### Balancing contradicting risk perceptions

Differences in their understandings of health, risk, and disease also seemed to have a strong influence on the participants’ perspectives of GDM and their approach to women and the recommendation of screening.

Some participants found that the guidelines for follow-up care conflicted with their professional values and beliefs, making them reluctant to discuss the increased risk of T2DM after GDM and the issue of routine screening:

*'The challenge is that these women are given an illness profile when they’re actually not ill. Erm … I feel a bit conflicted about this … because, yes, we must prevent disease and promote health, and we have to detect it early on and all that. But we certainly also need to be careful not to make them sick before they actually are. And that’s quite a balancing act.'* (C)

While none of the participants directly rejected the highly increased risk of T2DM during interviewing or opposed the idea of early detection, they were concerned about a general medicalisation of this group of young and supposedly healthy women. Some explained that they navigated this challenge by simply giving lower priority to this aspect of care; others said they struggled to find the right way to discuss this risk with women, without causing them unnecessary stress. In contrast, other participants espoused a more biomedical perception of risk, favouring screening while expecting women to have a high level of self-care by booking a screening:

*'If we have agreed that she* [the woman] *books a time, I’d expect her to do so. I won’t be keeping an eye on them to check if they’ve actually done it … because I mean, they are grown-ups — they must understand* [the risk] *… they do not suffer from dementia, unable to remember anything … so really, I’d actually not spend more time on that.'* (I)

This group expected women to take responsibility for their own health and access screening, although they generally acknowledged that a life with small children and work could be challenging.

Overall, many participants felt highly ambivalent about screening and communicating with women with previous GDM. Such barriers had led some to refrain from discussing the risk with women, while others found it difficult and a source of frustration in a busy everyday clinic.

### Theme 2: Prioritisation of early detection of diabetes

The participants generally supported efforts for early detection of diabetes in general practice. They found the supporting evidence solid and saw disease prevention as a significant element of their professional work:

*'This is our core competency as GPs … I expect that it is how we work … We were trained to look at risk factors … there’s absolutely nothing new in that.'* (I)

Most GPs saw disease prevention not only as a professional duty, but also as an important set of professional skills. Many GPs proudly expressed their belief in these skills as essential to their professional role, although they had often felt that circumstances had forced them to weaken their focus on these aspects of their clinical work. Their staff expressed similar views.

The insufficient focus on T2DM screening after GDM was often explained by reference to the urgent need to prioritise the most pressing problems in general practice. In some cases, the barriers to screening stemmed from the lack of resources with testing and an overload of tasks related to early detection of diabetes in general:

*'We run so many, many more tests for early detection of diabetes than for those who had GDM. Because it* [early diabetes detection] *is such a giant mountain of problems here, and women with previous GDM simply count next to nothing in that context.'* (F)

While the feeling of overload was particularly evident in solo practices, the larger group practices were also challenged. Some participants took an organisational perspective by arguing that the overall healthcare system was already at a tipping point. With an inherent fear of overburdening the system, some felt it was their duty to help prevent its breakdown by refraining from routine activities, among which they counted the screening of women with previous GDM.

### Theme 3: System influence on clinical procedures

#### Systematising clinical procedures to improve quality of care

Many of the participating GPs were open to increased systematisation and standardisation of procedures, as they found it improved their ability to make the necessary clinical judgements. They believed a systematised focus would facilitate the screening of women with previous GDM:

*'I think it’s about how well organised you are and what you focus on, making sure to say it’s a priority. Otherwise, it would be lost among all the other things in our busy everyday work. Because we’re … there’s so much to do … Over the last five years, we have become particularly attentive and got more routines for different things, including GDM women. I actually think it has made us better at our job as doctors.'* (B)

This approach also seemed to contribute to increased job satisfaction, as they believed it had improved their work and made it less stressful. The large group practices were particularly responsive to the healthcare authorities’ recommendations for systematising screening in their practices. In some cases, they had formulated internal guidelines to support their GPs and staff in transferring information for the benefit of the clinical work and to ensure that important knowledge was retained over time. Such guidelines seemed to benefit from local adjustments. In the most successful cases, the process relied on interdisciplinary or peer discussion and pragmatic compromising.

In a few of the most dedicated general practices, the record systems had been programmed to give pop-up reminders of screening to GPs or designated staff, a procedure they found had strongly improved screening routines. The facility also tracked women who cancelled or failed to make the expected booking for screening. However, the practical implementation of pop-up reminders appeared to be challenged by issues such as insufficient information transfer on women’s GDM diagnosis from the specialised healthcare sector. All participants shared a positive attitude to the use of screening reminders if they were handled outside general practice. Delegating the responsibility for systematic enrolment of patients and testing to practice staff appeared to facilitate screening; however, not all general practices had achieved this level of organisation and some GPs had no wish to delegate responsibility.

#### Trusting own clinical skills without system interference

Some GPs showed a less overt resistance to systematisation and standardisation of their procedures in the practice. They demonstrated an unwavering professional pride and a desire to preserve their independence and integrity, while relying on their ability to make clinical judgements in each individual situation:

*'We’re not happy about working directly to guidelines, because that’s not how our world is, anyway. I think you just adjust things according to your own ideas and your gut feeling … If you kind of know the overall guidelines … you just do what you think is best.'* (L)

Expressing their weariness or outright frustration, these participants generally considered national guidelines as interference on the part of healthcare authorities, while making clear their strong wish to protect their independence. As reported by participating staff members, some GPs preferred to rely on the knowledge accumulated over decades in the job. However, this often appeared to lead to differences in the offered care, occasionally resulting in barriers to follow-up of women with previous GDM. Compared with the GPs who sought to systematise work in their clinics, those who disapproved of what they saw as system interference seemed to be less inclined to update their knowledge. Almost all participants relied primarily on national public Danish healthcare service e-portals for updated information.

#### Influence of recommended test

The participants generally agreed that the recommended test for screening had increased uptake. They found the HbA1c test convenient for women and perceived it as an improvement that had eradicated previous barriers, such as the discomfort of the OGTT or that fasting was needed. While they appreciated that the hospital laboratory test results arrived on the same day, some GPs argued that the process could be further improved:

*'It would be great if we received the test results right away. Then we could look at them in relation to weight and blood pressure and see the bigger picture and how the woman is doing. The dialogue isn’t as good when they have to call in the next day … maybe one day we’ll have an* [in-house] *analyser.'* (E)

Despite differences of opinion, many saw the potential of performing in-house analysis, as this might improve the communication with women about their health.

## Discussion

### Summary

Having identified several facilitators of follow-up screening, this study found an acceptance of the overall responsibility for early detection of diabetes. However, even if GPs have a positive overall attitude towards early detection and prevention of disease, there are other factors they must take into account, such as time and resource availability. In follow-up screening among women with previous GDM, this involved challenges in addressing women’s risk, other pressing issues in clinical work, and barriers relating to standardised screening.

### Strengths and limitations

While the inclusion of both GPs and staff with delegated responsibility for the prevention of T2DM may be considered a limitation of this study, their different perspectives offered more nuanced data. The purposeful sampling strategy and the mixed sample reflect the diversity in Danish general practices, where postpartum consultations and screening for T2DM are often delegated to nurse or midwife staff. Accommodating a high level of information power, this sampling enabled the collection of richly varied in-depth data.^36^ However, inclusion of younger GPs with more recent qualifications or GPs with higher seniority in general practice may have influenced the results in a different way. With her background and professional mindset established outside general practice settings, the interviewer encouraged the participants to supply detailed descriptions. The methodological literature recommends this approach, in combination with the interviewer's prior understandings of the topic, as it is found to stimulate dialogue during data collection.^37^

### Comparison with existing literature

The most important barrier to follow-up screening of women stemmed from a hesitant, occasionally even reserved, approach to communication with the affected group. The finding is supported by previous studies documenting the reluctance among healthcare professionals towards addressing the particular risks faced by women with previous GDM.^38^ The present study's findings supplement previous research by elucidating possible underlying reasons, among which should be foregrounded the fact that in general practice, young and seemingly healthy women are not usually associated with risk. To this should be added that GPs and staff harbour ambivalent feelings and frustrations stemming from a risk perception in conflict with the national guidelines. This could help explain women’s experience that GPs give low priority to follow-up screening, providing women with little opportunity for elaboration and communication on risks and recommendations.^13,39^ The importance of addressing the underlying reasons for insufficient follow-up is emphasised in studies documenting that health professionals in general practice play a key role in women’s ability to fully understand the risk. Their support is thus crucial to women’s motivation to attend screening, in particular as exclusion from decisionmaking processes may lead to anxiety and uncertainty.^11^ Insufficient knowledge about the risks and benefits of screening of women was a further barrier to follow-up screening in general practice. The lack of awareness of guidelines seemed to partly explain this, while information about GDM diagnoses appeared to be lost in transition between the healthcare sectors.

The findings may partially be explained by the challenges of keeping updated on guidelines; a potential barrier was the discouragement felt by some GPs about their work being dictated by national guidelines. Their strong wish to make their own person-centred decisions seemed to contribute to the variation the study found in the priority given to follow-up screening. A related issue is the well-known difficulty of adopting evidence-based practices (EBP) in the primary health sector.^40^ However, it has been acknowledged that the rational, linear, and research-based approach recommended for EBP does not always fit into the messy reality of general practices, with staff experiencing stress from the need to juggle a growing mass of information and complex responsibilities.^40^

In addition to suggesting that the use of guidelines, most logically, facilitates follow-up-screening, the study has also offered new insights concerning its successful implementation. While the adoption of clinical guidelines may lead to quality improvements in some practices, others have experienced minimal effect.^41^ Where successful implementation of guidelines was identified, it was predominantly accompanied by interdisciplinary or peer discussions and local adjustments. The discussions stimulated by the GPs’ attention to women with previous GDM and follow-up screening involved not only their clinic colleagues, but also peers in other clinics and professional fora.

The study highlights the ever-present challenge of securing the effective sharing of information across healthcare sectors for successful follow-up screening.^11,13^ To strengthen communication and continuity of care, improving the discharge summaries from hospital sector to general practices is suggested, a solution that other studies have found helpful in improving continuity of care for women with previous GDM.^42^ ^16,38^

The authors saw the potential in the few cases where the clinics used their own record system to send pop-up reminders. The literature, however, suggests that this facility itself does not necessarily support the transition of patients across healthcare sectors.^42^ Danish GP clinics are offered a variety of commercial record systems and it is unclear if they all offer this feature; this may partially explain why pop-ups are not used on a larger scale. Another possible reason for the poor implementation and effect of pop-up reminders in general practice might be that the delivery of such reminders seemed to depend on sufficient information sharing across healthcare sectors, as found in the present study.

Although the findings support earlier studies, indicating that routine screening activities of women with prior GDM are frequently given low priority,^11,36^ the available screening test (HbA1c) was found to facilitate follow-up screening. The authors believe that its general introduction could help minimise the barriers to participation faced by women, such as time expenditure and the discomfort of the OGTT.^11^

### Implications for practice

GPs are called to familiarise themselves with the guidelines as it was seen that this facilitated uptake of follow-up screening. Professional collaboration and adaptation support has also been shown to lead to successful implementation of guidelines in general practice. This includes interdisciplinary or peer discussion, debate, and knowledge sharing to support adjustments in the individual practices. This could help GPs identify procedures suited to their resources and organisation, as well as target the underlying reasons for insufficient follow-up, which was related to women posing an often unfamiliar risk. By ensuring the involvement of all parties in the process, the familiarisation of guidelines and possible implementation can promote continuity of care for women, while alleviating some GPs’ frustrations about external recommendations. The initiatives should, however, not be restricted to the implementation of guidelines, but also aim to support GPs and their staff’s reflection and discussion of ways to engage in communication and decision-making processes with women, the lack of which was found to create an important barrier to follow-up screening. These findings could help inform the development of future interventions, such as the use of electronic reminders, as it provides important knowledge for intervention modelling and adaptation processes.

## Data

The dataset relied on in this article is available from the corresponding author on reasonable request.
